# Customization of Stock Eye Prosthesis for a Pediatric Patient by a Simplified Technique

**DOI:** 10.5005/jp-journals-10005-1157

**Published:** 2012-08-08

**Authors:** Sunit Kumar Jurel, Naina Talwar, Pooran Chand, Raghuwar D Singh, Durga Shanker Gupta

**Affiliations:** Assistant Professor, Department of Prosthodontics, Faculty of Dental Sciences, Upgraded KGMC, Lucknow, Uttar Pradesh, India, e-mail: dentistmj1110@yahoo.co.in; Postgraduate Student (3rd Year), Department of Prosthodontics Faculty of Dental Sciences, Upgraded KGMC, Lucknow, Uttar Pradesh, India; Associate Professor, Department of Prosthodontics, Faculty of Dental Sciences, Upgraded KGMC, Lucknow, Uttar Pradesh, India; Assistant Professor, Department of Prosthodontics, Faculty of Dental Sciences, Upgraded KGMC, Lucknow, Uttar Pradesh, India; Senior Lecturer, Department of Oral and Maxillofacial Surgery Teerthanker Mahaveer Dental College and Research Centre Moradabad, Uttar Pradesh, India

**Keywords:** Staphyloma, Enucleation, Ocular defect, Customized stock eye

## Abstract

The unfortunate loss or absence of an eye may be caused by congenital defect , irreparable trauma, tumor or blind eye. The role of the maxillofacial prosthodontist in fabricating an ocular prosthesis to restore facial symmetry and normal appearance for the anophthalmic patient becomes essential. A custom-made ocular prosthesis is an excellent alternative for the people who lose their eye especially in young age. It has acceptable fit, retention and esthetics but is technically difficult to fabricate. On the other hand the stock eye has compromised fit and poor esthetics. Our case report presents a simple technique of customization of stock eye prosthesis to provide accurate fit and acceptable esthetics.

**How to cite this article:** Jurel SK, Talwar N, Chand P, Singh RD, Gupta DS. Customization of Stock Eye Prosthesis for a Pediatric Patient by a Simplified Technique. Int J Clin Pediatr Dent 2012;5(2):155-158.

## INTRODUCTION

Several ocular and orbital disorders require surgical intervention that may result in ocular defects. Depending on the severity of situation, the surgical management may include one of the three approaches: Evisceration, enucleation or exenteration.^1^ Evisceration is the surgical procedure wherein the intraocular contents of the globe are removed, leaving the sclera. Enucleation is the surgical removal of the globe and a portion of optic nerve from the orbit. Exenteration is an en bloc removal of the entire orbit, usually involving partial or total removal of the eyelids, and is performed primarily for the eradication of malignant orbital tumor.^2^

The associated psychological effect of these defects on the patients require immediate management and rehabilitation of the defect by a team of specialists. This requires a collaborated effort of plastic surgeon, ophthalmologist and maxillofacial prosthodontist. This article presents a simplified technique of rehabilitation of ocular defect in 11-year-old girl.

## CASE REPORT

**Fig. 1:**
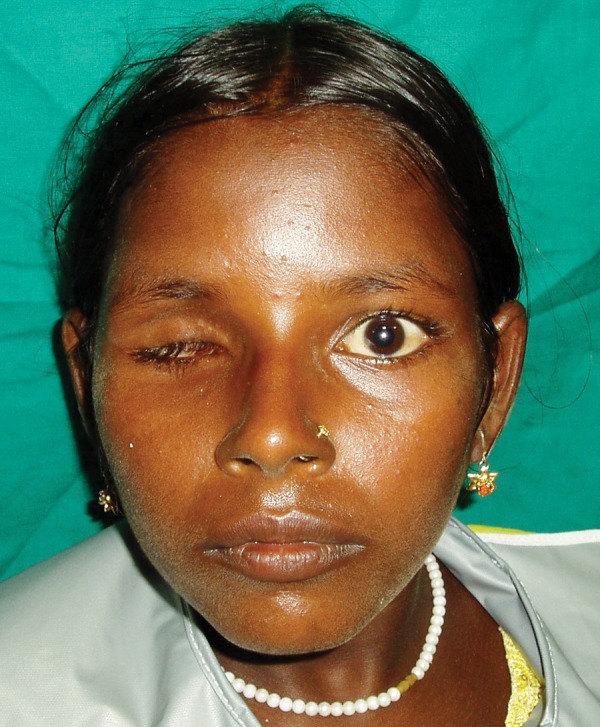
Pretreatment view showing the anophthalmic patient

An 11-year-old female patient was referred to the Department of Ophthalmology, CSMMU with the chief complaint of pain, bulging and whitish appearance of the right eye. She was diagnosed with anterior staphyloma for which the enucleation was done. The patient was then referred to the Department of Prosthodontics, CSMMU for the fabrication of eye prosthesis. On examination the defect area was found asymptomatic (Fig. 1) and it was decided to fabricate an ocular prosthesis. The treatment plan was explained to the patient and a written informed consent of the patient was taken.

## TECHNIQUE

**Fig. 2:**
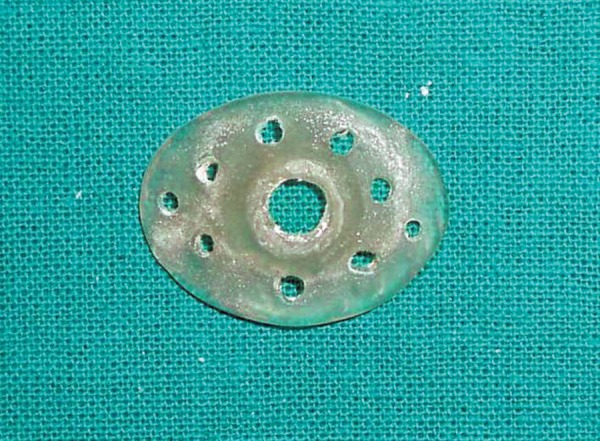
Acrylic resin custom tray with perforations and an injection hole at the papillary position

1. A stock eye whose iris and pupil matched with the patient's contralateral eye was selected and was made to fit the anophthalmic socket by trimming it till it was loosely fitting and comfortable to the patient.
2. The eye was then invested in irreversible hydrocolloid (Vignette-chromatic, Dentsply India) and a mold is made. After the material is set the eye shell was removed and replaced with self-cure autopolymerizing acrylic resin to obtain a custom ocular tray. Perforations were made in the tray and a tunnel was made through the center at the approximate positioning of the pupil (Fig. 2).
3. An injection tube was fabricated from a 5 ml plastic syringe. The needle was removed from the syringe and its cap was cut approximately half way from the end. Its edges were roughened and wedged into the pupil perforation hole. The tray was cleaned and disinfected. Barrel of the 5 ml syringe was attached to the injection tube. It was then tried in the socket to check for overextension and proper orientation (Figs 3A to C).
4. Ophthalmic alginate (Ophthalmic moldite, Milton Roy Co. Sartosa Florida) wax mixed with an extra half of warm water to obtain a smooth, runny mix. Anophthalmic socket and surrounding area was coated with a lubricant (petroleum jelly) to prevent the impression material from sticking to it. Upper and lower eyelids were retracted and at the same time syringe was backloaded by the assistant. The plunger was inserted, tray was seated in position and the material was injected through the syringe. The patient was instructed to make all eye movements to allow the material to flow into all the areas. After the material was set the impression was removed from the shallower lower sulcus first and then rotated from the deeper sulcus. It was then examined for voids (Fig. 4).
5. A two piece cast was made in dental stone to obtain a positive replica of the posterior aspect of the eye. This was done by pouring the impression in two parts with the second part being poured after applying separating media and making orientation grooves (Fig. 5A).
6. Wax pattern of the sclera was obtained by flowing liquid modeling wax mixed with inlay wax (Figs 5A and B). The previously selected stock eye was made to fit the wax pattern and adapted to the cast. The wax pattern- stock eye assembly was seated in the socket and facial measurements were made to check the position of iris and pupil (Fig. 6). After correct papillary alignment, palpebral movement, sclera contour and convexity and eye closure was checked. The eyelids should close completely over the wax pattern-stock eye assembly.
7. Scleral shade was selected by mixing small amounts of tooth colored acrylic and matching with contalateral eye. Then the adjusted stock eye-wax pattern assembly was invested, flasked and dewaxing was done. Red silk fibers to mimic viens were placed in the dough of the predetermined acrylic shade followed by routine packing, curing, finishing and polishing. The anterior sclera curvature was reduced approximately 1 mm to add a thin film of transparent heat cured PMMA (Trevalon, dentsply Pvt Ltd, Gurgaon, India) to copy corneal translucency (Fig. 7).
8. The properly finished and polished prosthesis was inserted into the socket after being disinfected and was lubricated with an ophthalmic lubricant to maintain a thin film of tears over the prosthesis and to improve eye movements (Fig. 8).
9. The prosthesis was delivered and instructions for insertion and removal of prosthesis were given, cleaning and the need for cleaning of prosthesis and regular recall appointments was emphasized.

**Fig. 3A:**
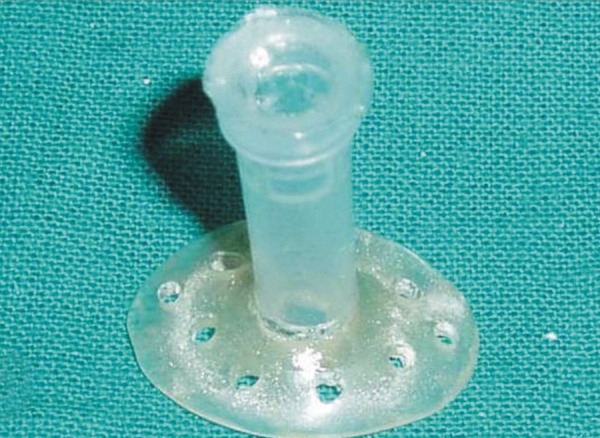
The syringe cap cut halfway and attached to the custom tray

**Fig. 3B:**
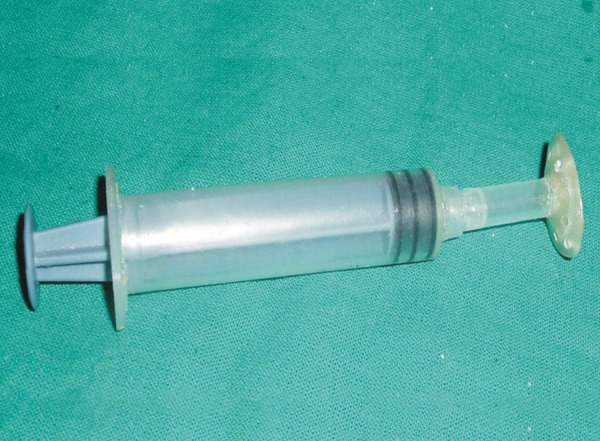
The tray is screwed into the syringe barrel

**Fig. 3C:**
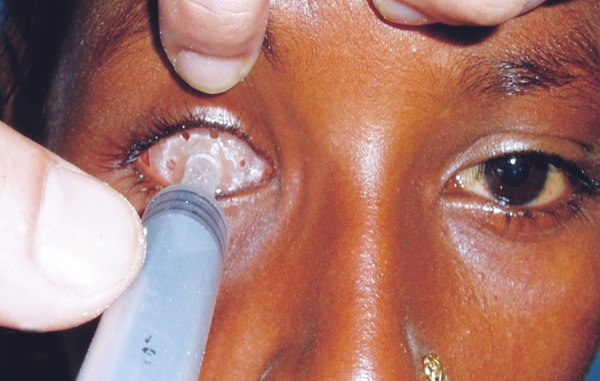
Tray with injection tube tried in the eye socket

**Fig. 4:**
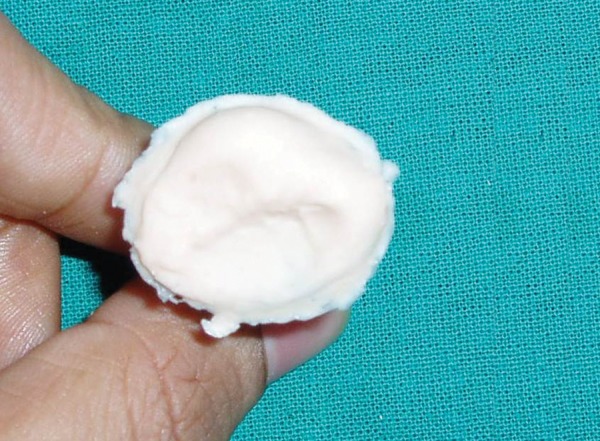
The resulting impression

**Fig. 5A:**
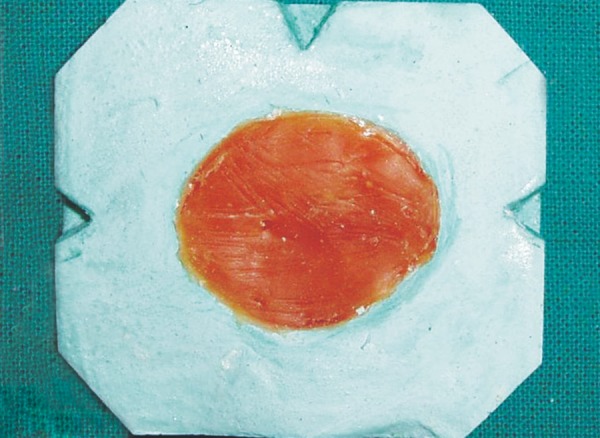
Two piece cast

**Fig. 5B:**
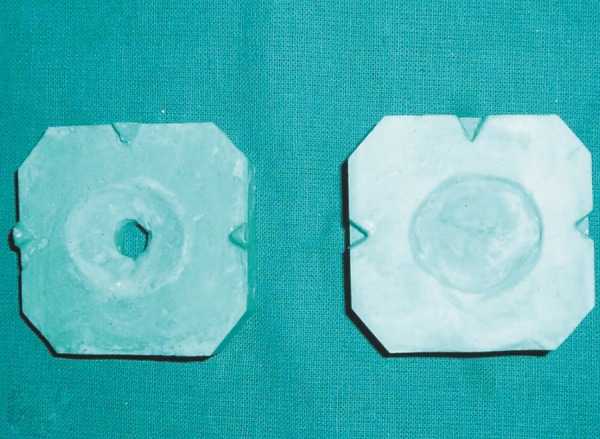
Scleral wax pattern

**Fig. 6:**
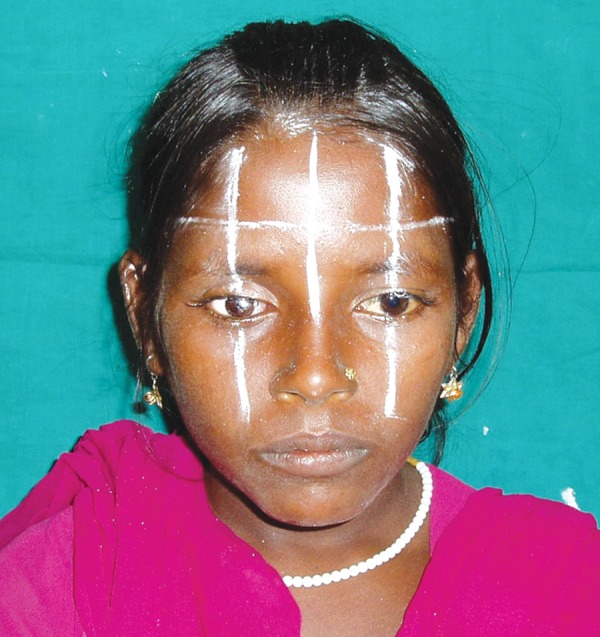
Wax pattern try in with facial measurements to check the position of pupil and iris

**Fig. 7:**
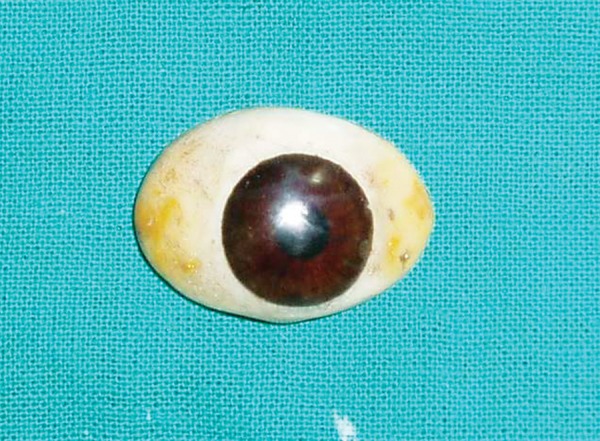
Finished prosthesis with characterization

## DISCUSSION

The disfigurement associated with the loss of an eye can cause physical and emotional problems.^3^ The occurrence of this defect in childhood can have a great impact on the growth and development. It can lead to embarrassment and social withdrawal. Hence, early rehabilitation of this defect is of paramount importance to promote physical and psychological healing of the patient and social acceptance.

The need for artificial eye can sometimes be satisfied by stock prosthesis that come in standard sizes, shapes and colors.^4-6^ These are relatively inexpensive and can be delivered quickly. But, their disadvantages include poor fit, compromised esthetics and poor eye movements. The poor fit can lead to complications, such as granuloma formation.^4,7-9^ Thus, their use can be limited for interim purpose only. Often however, a custom ocular prosthesis is indicated.^6-8,10,11^ The custom, made ocular prosthesis has improved adaptation to underlying tissues, increased mobility of prosthesis, improved facial contours, and enhanced esthetics due to control over the size of iris and pupil and color of the iris and sclera.^8,9,12-14^ But, this involves complex painting procedures and high skill and expertize of the patient. At the same time it is more expensive.^1,7,13^

In our technique we have customized the stock eye to gain accurate fit and acceptable esthetics by a simple technique. This helped us to overcome the disadvantages of poor fit and compromised esthetics of stock eye and complex and precise painting procedures of custom-made eye. This technique does not depend on the artistic ability of the operator and is easy. The close adaptation of the prosthesis to the tissue bed provides maximum comfort and restores full physiologic function to the accessory organs of the eye. Voids that collect mucus and debris, which can irritate mucosa and act as a potential source of infection can also be minimized and this prosthesis provides optimum cosmetic and functional results. Although an implant retained prosthesis is superior in terms of simulating eye movements it was not planned because of the young age and economic reasons.

**Fig. 8:**
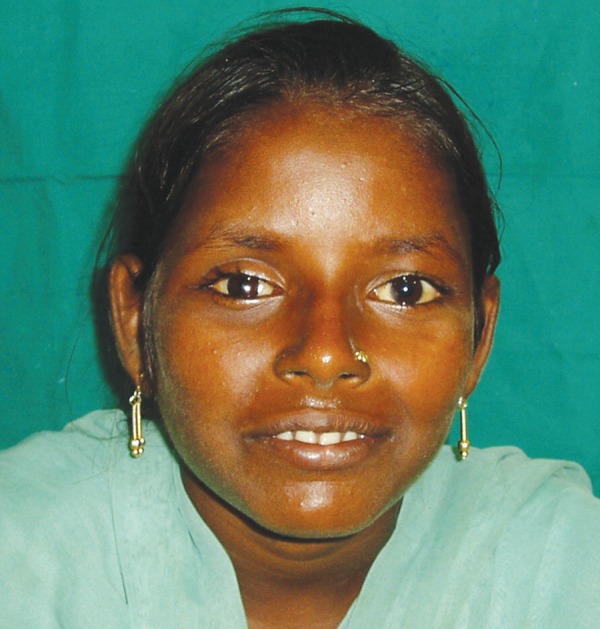
Posttreatment view with prosthesis in place

The only disadvantages of the technique is the availability of properly matching color of iris of the stock eye. Also the patient may have to be kept on recall to monitor the color stability of the relined prosthesis.

## CONCLUSION

A relatively easy and inexpensive technique of customization of stock eye has been presented. The success of this technique depends on an accurate impression of the eye socket and on the availability of a closely matching stock eye. Although the patient cannot see with the prosthetic eye, it can restore her back her confidence and esthetics.
